# Innovative treatment strategies for impacted maxillary central incisor and case comparisons: Case report

**DOI:** 10.1097/MD.0000000000049675

**Published:** 2026-08-14

**Authors:** Wei Chen, Jingyao Bai, Wei Zhang, Jiqin Zhang

**Affiliations:** a The First People’s Hospital of Linping District, Hangzhou, Zhejiang, China; b Department of Orthodontics, Hefei Stomatological Hospital, Hefei, Anhui, China; c The Third Affiliated Hospital of Zhejiang Chinese Medical University (Zhongshan Hospital of Zhejiang Province), Hangzhou, Zhejiang, China.

**Keywords:** mimics software, root development, root surface area, root-oriented orthodontic traction treatment, root volume

## Abstract

**Background:**

The present article compares innovative treatment strategies for impacted maxillary central incisors, emphasizing the critical importance of prioritizing root development over rapid crown alignment.

**Methods:**

Two case reports using distinct clinical approaches are presented. Case 1 utilized a traditional combination of closed-eruption surgical exposure and conventional orthodontic traction, while Case 2 utilized a novel root-oriented traction technique, which ensures the development of tooth roots and utilizes the natural eruption power.

**Results:**

Both techniques achieved successful eruption and alignment. However, the root-oriented method demonstrated superior outcomes in promoting continued root formation, manifested as a larger volume and surface area of tooth roots, reducing the risk of premature apical closure.

**Conclusion:**

The primary objective in managing an impacted maxillary central incisor should be to reduce root developmental resistance and ensure substantial root development, rather than to achieve rapid crown alignment.

## 1. Introduction

The failed eruption of maxillary incisors not only compromises dentofacial aesthetics but may also lead to reduced self-esteem.^[1]^ Impacted maxillary central incisors typically manifest in the early mixed dentition, arising from multiple potential etiologies.^[2]^ These include supernumerary teeth in the anterior maxilla, agenesis of the affected incisor, delayed resorption of the anterior deciduous teeth, root dilaceration secondary to trauma, or impaction due to local pathology such as cysts.^[2–4]^

When dealing with impacted maxillary central incisors, a composite of clinical strategies is utilized, including post-extraction restoration, autogenous tooth replantation^[5]^, and orthodontic traction facilitated by surgical intervention. Although studies have corroborated the efficacy of amalgamating surgical and orthodontic traction as a therapeutic pathway,^[6]^ it often necessitates direct extraction or post-traction treatment extraction due to the high prevalence of curved roots and a bleak prognosis.

Through a comparison of conventional orthodontic traction and a novel root-oriented traction technique,^[7]^ this study investigates whether guiding the root of the impacted tooth to a position free of developmental resistance facilitates its maturation, thereby informing a more scientific treatment strategy for managing impacted teeth.

## 2. Methods

The patients were recruited from June 01, 2023 to March 31, 2025, during which the entire treatment process was completed. Written informed consent from the guardians was obtained before the treatment.

Axial cone-beam computed (CBCT) digital imaging and communications in medicine images were imported into Mimics. Initial segmentation was performed using thresholding (620-3517 HU) to isolate dental hard tissues. The region-growing tool was applied to isolate individual teeth. Subsequently, extensive manual editing of the mask was undertaken in all planes using the Edit Mask and Split Mask tools to meticulously eliminate any redundant tissue with adjacent teeth or bone, ensuring an accurate representation of the target tooth’s geometry. Following three-dimensional (3D) model reconstruction, the software’s integrated calculation tools were used to directly compute the volume (mm^3^) and surface area (mm^2^) from the high-fidelity mesh. All measurements were performed in triplicate by a single blinded operator to ensure reliability.

### 2.1. Case 1

#### 2.1.1. Medical history

A 7-year-old male patient presented for evaluation of delayed eruption, exceeding 6 months’ duration, in tooth 21 and partial eruption of tooth 22, with clinical assessment revealing incomplete coronal emergence of tooth 11. The patient maintained good systemic health with an unremarkable systemic disease history and no documented history of dental anomalies or maxillofacial trauma.

#### 2.1.2. Examination

Intraoral examination revealed a mixed dentition pattern characterized by retained primary teeth (53, 54, 55, 63, 64, 65, 73, 74, 75, 83, 84, 85) and erupted permanent dentition (11, 16, 22, 26, 31, 32, 36, 41, 42, 46). Carious lesions were detected in multiple primary molars (55, 64, 65, 75, 84, 85). Notable findings included congenital absence of primary maxillary lateral incisors (52, 61), accompanied by a mesial inclination of permanent central incisors (11, 22), creating a diastema of 3 mm. Occlusal analysis demonstrated bilateral Angle Class I molar relationships with physiologically acceptable vertical dimensions (overjet: 2 mm, overbite: 2 mm). Palpation of the maxillary buccal sulcus showed normal anatomical contours without detectable swelling or cortical expansion (Fig. 1).

**Figure 1.**
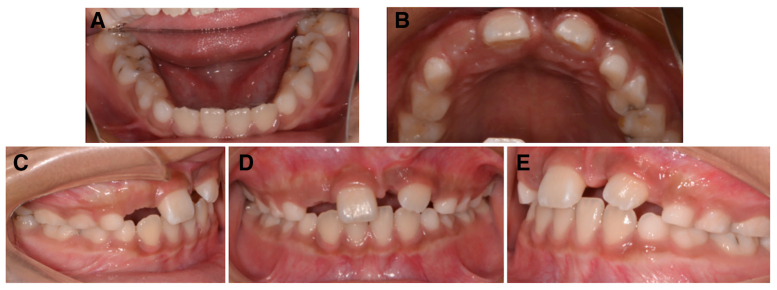
Intraoral photographs pre-operation. (A–E) The absence of tooth 21 accompanied by a mesial inclination of tooth 11 and tooth 22.

#### 2.1.3. CBCT analysis

CBCT images revealed a highly inverted impacted tooth 21, with preservation of the surrounding alveolar bone volume. Root development corresponded to Nolla stage 7 to 8 (Fig. 2).

**Figure 2.**
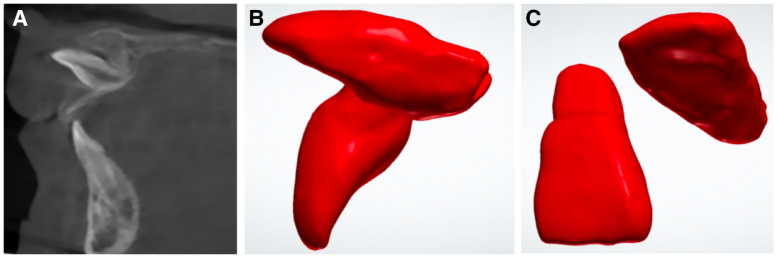
CBCT images and 3D reconstruction in Mimics software pre-operation. (A) Revealed a high inverted impaction of tooth 21; (B, C) The development of the root was classified as Nolla stage 7–8. 3D = three-dimensional, CBCT = cone-beam computed.

#### 2.1.4. Diagnosis

Inverted impacted tooth 21.

#### 2.1.5. Treatment options discussion

Following a comprehensive review of potential treatment strategies, including autogenous tooth replantation, extraction with subsequent fixed restoration, surgical exposure, and orthodontic traction, the decision was made by the patient’s guardians to pursue a combined approach of surgical exposure and orthodontic traction treatment.

#### 2.1.6. Treatment progress

In the first step of treatment, the patient has already had a customized active orthodontic device installed to align the erupted teeth. Afterwards, oral surgery was performed. Under local anesthesia, a closed eruption technique was employed. The crown of tooth 21 was revealed by an access window (Fig. 3A). Minimal removal of the mucoperiosteum and dental follicle was performed to facilitate bracket bonding. Hemostasis was achieved using adrenaline-soaked cotton strips, followed by etching of the tooth’s surface, attachment of a bracket via a 0.25 mm ligature wire-formed traction hook, and wound closure to maintain the hook’s exposure (Fig. 3B).

**Figure 3.**
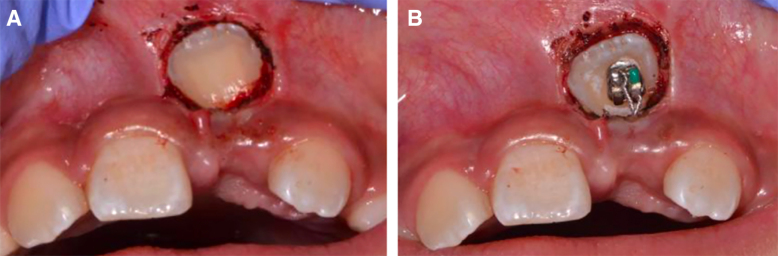
Closed eruption technique for impacted tooth 21. (A) Creating an access window for the crown of tooth 21; (B) Attachment of a bracket via a 0.25 mm ligature wire-formed traction hook.

During a 10-day post-operative assessment, the patient was observed utilizing the active orthodontic apparatus, with the protruding traction hook through the periosteum linked to another hook on the adjustable orthodontic device using an elastic band. This band was replaced daily, alongside adjustments to the gap spring to concurrently initiate traction and gap creation for the missing tooth. The patient underwent evaluations every 3 to 4 weeks to monitor and adjust the traction’s force and direction by altering the hooks on the adjustable device. Regular monthly follow-ups were scheduled, during which the positioning of certain traction hooks was modified to optimize the direction and force of traction. The adjustment of the gap spring was also periodically reassessed. Three months post-operation, tooth 21 erupted (Fig. 4A and B). At this juncture, the lingual metal bracket on tooth 21 was replaced with a lingual button affixed to the buccal crown side. Six months post-emergence, half of tooth 21 crown had integrated into the dental arch, necessitating the use of a 2 × 4 straight wire fixed orthodontic appliance (Fig. 4C). By the seventh month, the lingual button was substituted with a metal bracket, noting that the nickel-titanium wire had not been fully seated within the bracket, being temporarily secured by the ligature wire alone (Fig. 4D). At the eighth month review, the nickel-titanium wire was successfully ligated within the bracket (Fig. 4E). By the ninth (Fig. 4F) and tenth months (Fig. 4G), tooth 21 had gradually aligned within the dental arch, which was fully accomplished at the eleventh month (Fig. 4H). Treatment concluded 14 months post-operation, marking the removal of the bracket and the installation of a lingual retainer wire. Clinical examinations revealed no signs of gingival redness, swelling, or probing bleeding, with a periodontal probing depth between 1 to 2 mm, albeit noting an absence of keratinized gingiva (Fig. 5). Pulp vitality tests yielded values of 37 for the treated side compared to 30 on the contralateral side. CBCT and mimics reconstruction revealed the curved but closed apical foramen (Fig. 6A–C). Mimics measurement software was utilized for three-dimensional reconstruction and analysis, indicating an increase in the volume of the impacted tooth 21 from 413.4 to 475.2 post-operation, marking a volume enhancement of 61.8. Meanwhile, the surface area expanded from 439.7 to 507.6, reflecting a surface area enhancement of 67.9 (Fig. 7).^[8]^

**Figure 4.**
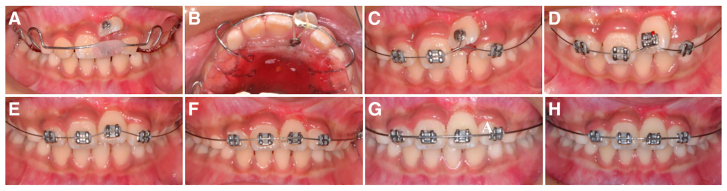
Post-operative intraoral photographs. (A, B) Three months; (C) Six months; (d) Seven months; (e) Eight months; (f) Nine months; (g) Ten months; (h) Eleven months.

**Figure 5.**
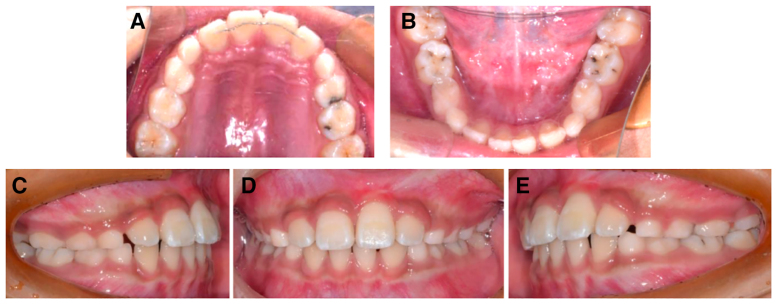
Intraoral photographs at 14 mo post-operation.(A–E) Clinical examinations revealed no signs of gingival redness.

**Figure 6.**
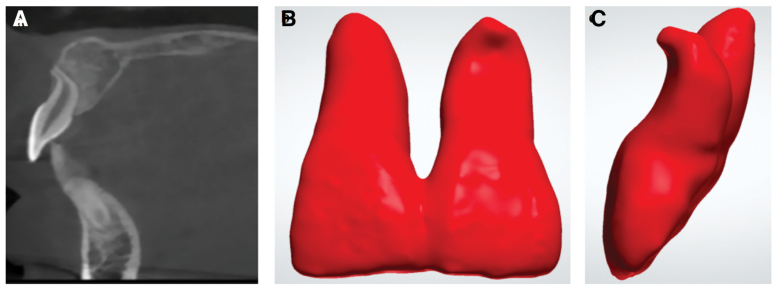
CBCT image and 3D reconstructions in Mimics software post-operation. (A) CBCT image post-operation shows the closed apical foramen; (B, C) Mimics reconstruction post-operation shows the curved root. 3D = three-dimensional, CBCT = cone-beam computed.

**Figure 7.**
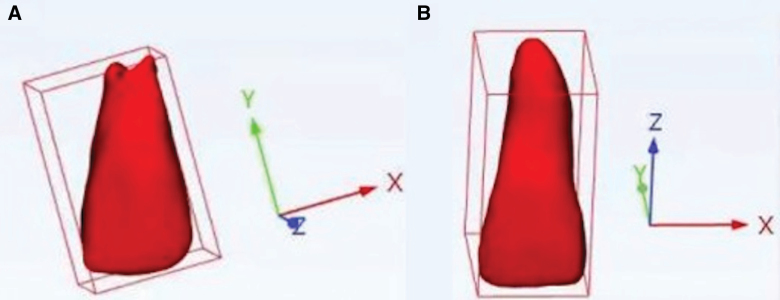
Analysis in Mimics software. (A) The volume of the impacted tooth pre-operation was 413.4 and the surface area was 439.7; (B) The volume of the impacted tooth post-operation was 475.2 and the surface area was 507.6.

### 2.2. Case 2

#### 2.2.1. Medical history

A female patient presented for evaluation of delayed eruption, exceeding 6 months in duration, in tooth 11, denying any systemic diseases or maxillofacial trauma.

#### 2.2.2. Examination

Maxillofacial examination revealed bilateral symmetry and incomplete upper lip fullness. Intraoral assessment indicated a mixed dentition stage, with the retention of deciduous tooth 51 and the absence of eruption of tooth 11, whereas tooth 21 had erupted. Notably, carious lesions were detected in multiple primary molars (75, 84, 85), and the anterior teeth showed mild overjet and overlap (Fig. 8).

**Figure 8.**
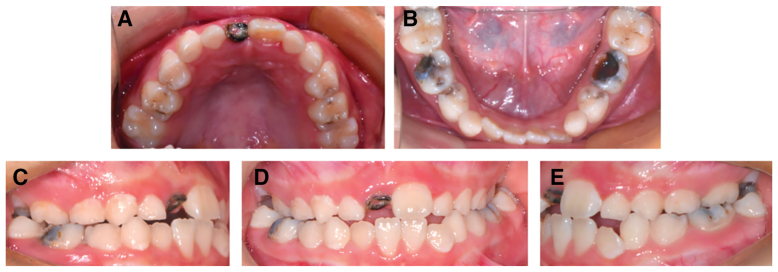
Intraoral photographs pre-operation. (A–E) Intraoral photographs show the retention of deciduous tooth 51 and the absence of eruption of tooth 11.

#### 2.2.3. CBCT analysis

Images revealed the significant inverted impacted tooth 11, characterized by an impaction angle of approximately 120 degrees. Periapical alveolar bone volume was deemed acceptable, with root development classified at Nolla stages 6 to 7 (Fig. 9).

**Figure 9.**
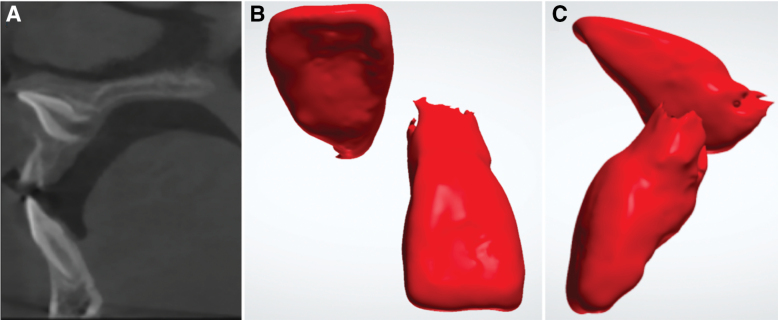
CBCT image and 3D reconstructions in Mimics software pre-operation. (A) CBCT image pre-operation shows the inverted impacted tooth 11; (B, C) Mimics reconstructions show the root development classified at Nolla stages 6–7. 3D = three-dimensional, CBCT = cone-beam computed.

#### 2.2.4. Diagnosis

Inverted impacted tooth 21.

#### 2.2.5. Treatment options discussion

Several treatment modalities were evaluated, including autogenous tooth replantation, extraction with subsequent fixed restoration, surgical exposure, and orthodontic traction. Autogenous tooth replantation was ruled out due to the advanced curvature of the root and incomplete root development. Considering the young age of the patient and the possibility of severe alveolar bone loss at the extraction site, coupled with concerns over aesthetics and the child’s mental health during the waiting period for growth, it has been decided to proceed with surgical exposure of the impacted maxillary central incisor followed by orthodontic traction treatment.

#### 2.2.6. Treatment plan

Stage 1: Combined eruption surgical technique with the use of an adjustable movable retractor for retracting tooth 11 into alignment within the dentition.
Stage 2: Application of a 2 × 4 technique for alignment of tooth 11.

##### 2.2.6.1. Stage 1

**Pre-operative and operative details**: The oral surgery involved the extraction of tooth 51, followed by window creation for crown exposure of tooth 11 and installation of a lingual clasp on the lateral aspect of the crown. A 0.025 mm metal wire was attached to the lingual clasp, forming a traction hook connected via a rubber band to the W-curve on the adjustable movable retractor. This allowed for gradual traction of tooth 11, with the retraction device being removable during meals (Fig. 10).

**Figure 10.**
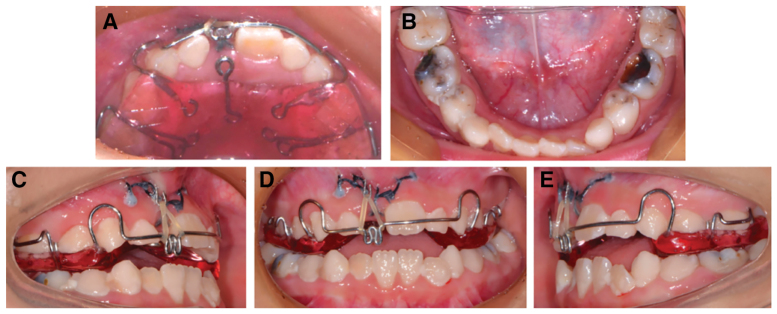
Window creation and installation of a lingual clasp for tooth 11. (A–E) Installation of a lingual clasp on the lingual side of tooth 11 and application of the retraction device.

Post-operative progress: Continuous development of the tooth’s root was observed without significant alveolar bone resorption (Fig. 11). Subsequent intraoral examination showed approximately one-quarter of tooth 11’s crown erupting labially, positioned at the maxillary vestibular groove. The metal lingual clasp was then replaced with a buccal side metal bracket (Fig. 12).

**Figure 11.**
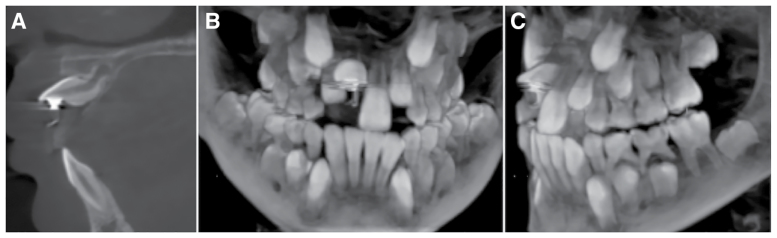
CBCT images showed the continuous development of the tooth’s root. (A–C) Continuous development of the root of tooth 11. CBCT = cone-beam computed.

**Figure 12.**
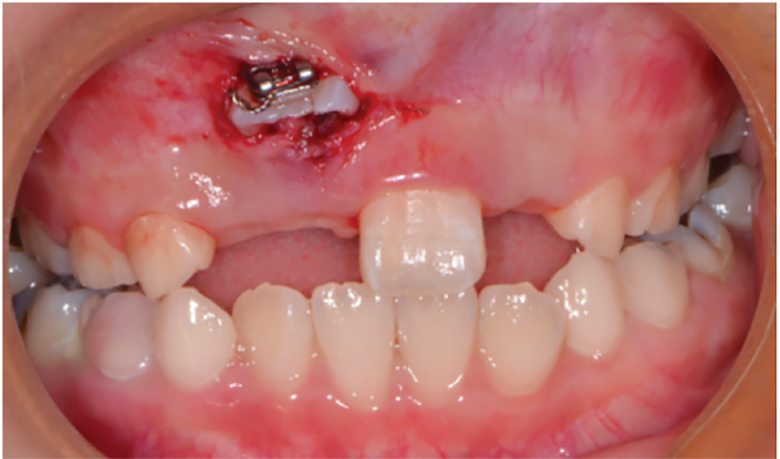
The metal lingual clasp was replaced with a buccal side metal bracket.

**Traction outcome**: After 10 months, half of tooth 11’s crown had erupted, displaying healthy gingiva in color and texture without crown discoloration (Fig. 13). Further examination revealed two-thirds of the clinical crown exposed buccally, aligning flush with tooth 21 (Fig. 14). The inversion correction was successful, reaching Nolla stage 7 to 8, with the apical foramen open and expanded, showing no notable resorption of adjacent alveolar bone (Fig. 15).

**Figure 13.**
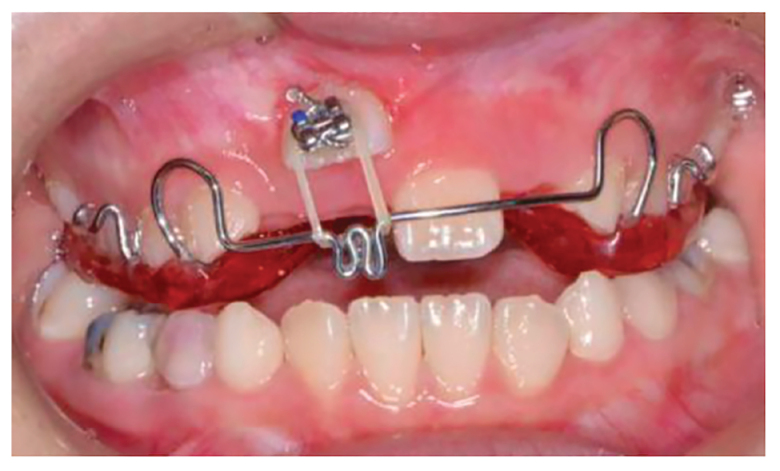
Ten months post-operation.

**Figure 14.**
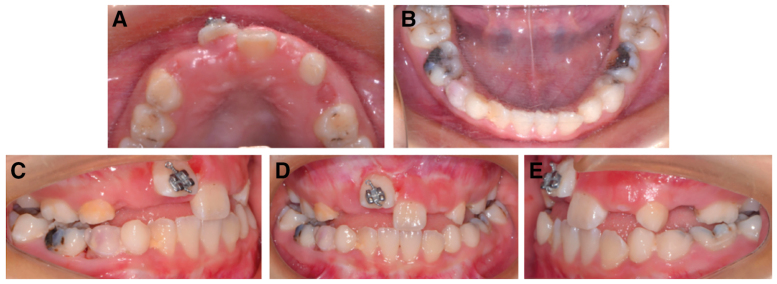
Intraoral photographs post-operation. (A–E) Two-thirds of the clinical crown exposed.

**Figure 15.**
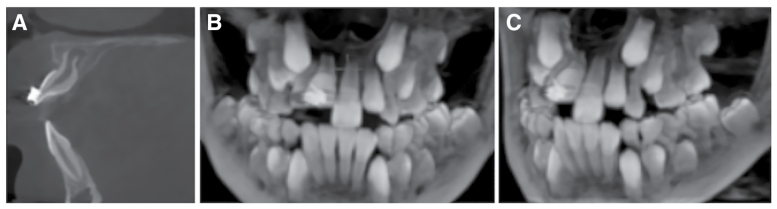
CBCT images post-operation. (A–C) The development of the root was classified as Nolla stage 7–8. CBCT = cone-beam computed.

**Follow-up observations**: At 16 months follow-up, tooth 11 was fully visible in the oral cavity, erupting buccally to align with the incisive edge of tooth 21 (Fig. 16). At the 27-month follow-up, stable eruption and positioning were confirmed, with normal gingival coloration and no recession (Fig. 17).

**Figure 16.**
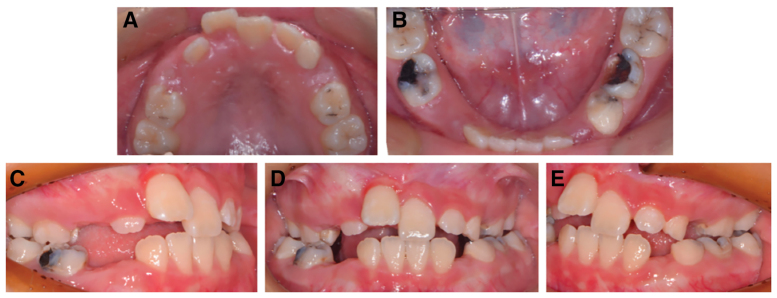
Sixteen months post-operation. (A–E) Tooth 11 continued to erupt.

**Figure 17.**
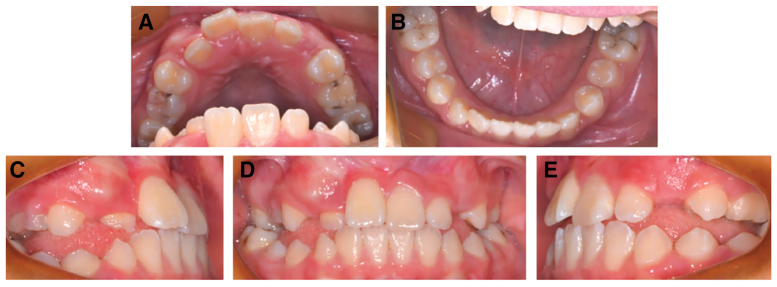
Twenty-seven months post-operation. (A–E) Stable eruption and positioning of tooth 11.

##### 2.2.6.2. Stage 2

The 2 × 4 traction technique was applied for the final positioning of tooth 11, secured with a lingual retention wire (Fig. 18). Comparison between pre-operative and post-operative data highlighted the significant improvement and alignment achieved when utilizing root-oriented orthodontic traction treatment (Fig. 19).

**Figure 18.**
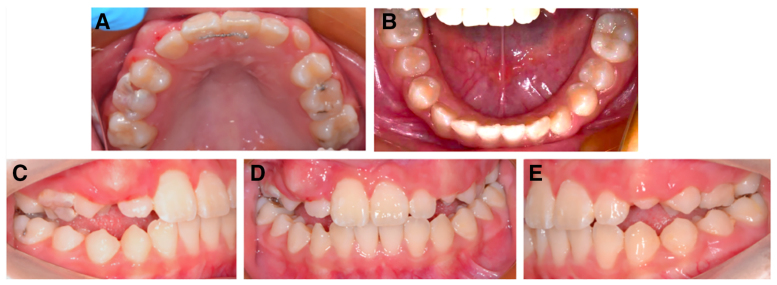
Treatment of stage 2. (A–E) The 2 × 4 traction technique was applied for the final positioning of tooth 11.

**Figure 19.**
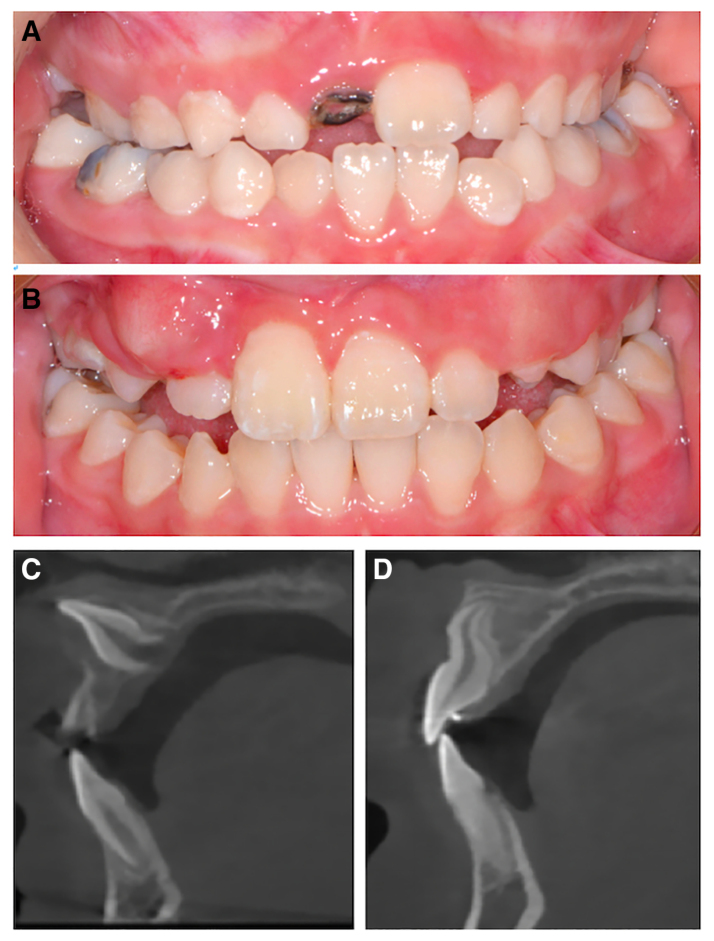
Pre-operation and post-operation comparisons. (A, B) Pre-operation intraoral and CBCT images; (C, D) post-operation intraoral and CBCT images. CBCT = cone-beam computed.

The 3D reconstruction of the impacted teeth in Mimics showed an increase in the volume of the impacted tooth 11 from 384.4 to 457.5 post-operation, marking a volume enhancement of 73.1. Meanwhile, the surface area expanded from 416.2 to 485.7, reflecting a surface area enhancement of 69.5 (Fig. 20).

**Figure 20.**
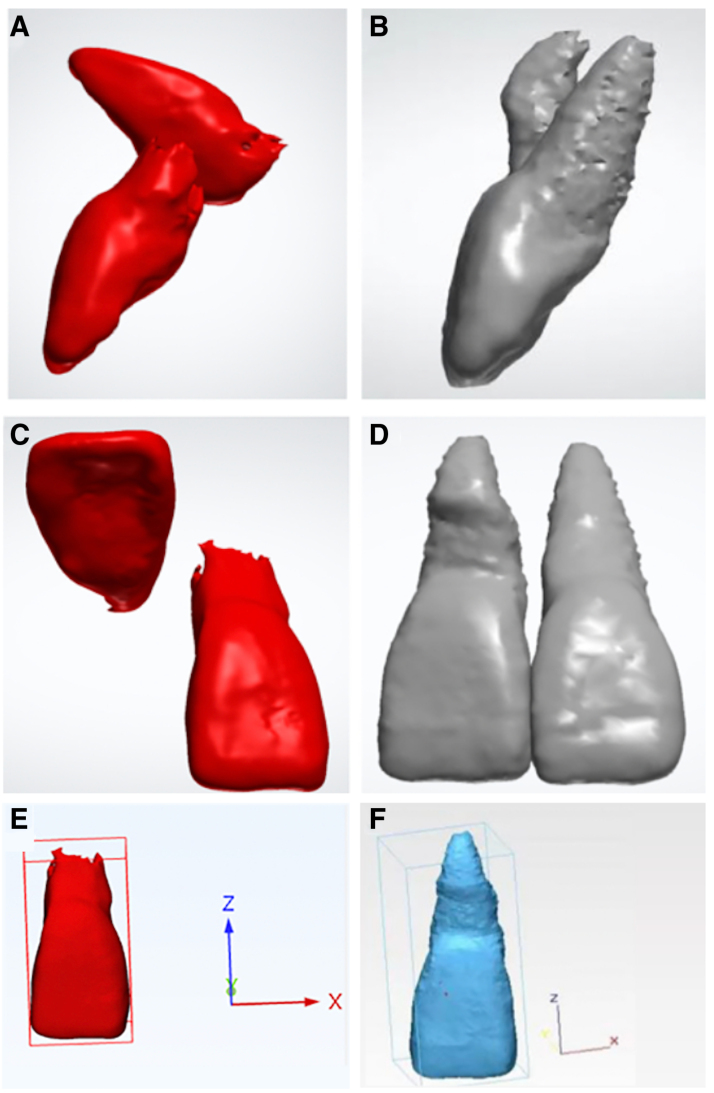
3D reconstruction and analysis in Mimics software. (A, C) 3D reconstruction pre-operation; (B, D) 3D reconstruction post-operation; (E) The volume of the impacted tooth pre-operation was 384.4 and the surface area was 416.2; (F) The volume of the impacted tooth post-operation was 457.5 and the surface area was 485.7. 3D = three-dimensional.

## 3. Discussion

Inverted impacted tooth presents a unique challenge in root development, usually resulting in malformation due to interference between the unmineralized and mineralized parts around the root. Sun et al have demonstrated that the early teeth’s impedance center, situated near the cervical area, predisposes them to rotational discrepancies.^[9]^ Maximal growth potential exists when the tooth root is approximately two-thirds formed (Nolla stage 8), advocating for the application of traction during this critical period. Additionally, initiating traction at an early stage for maxillary inverted impacted teeth not only facilitates root development but also minimizes the resorption of labial alveolar bone. Furthermore, the CBCT images have illustrated that teeth exhibit growth potential prior to apical foramen closure.^[9]^ The current study demonstrated that when the root of the impacted maxillary central incisor was pulled to a moderate position of the alveolar bone, even if the traction was stopped, the crown would continue to erupt due to root development. Utilizing Mimics 3D reconstruction, our study displayed a remarkable increase in tooth volume and surface area post-operation. Predominantly due to the ability of tooth roots to sustain development, early orthodontic traction significantly aids in creating space and directing the impacted tooth towards the correct eruption path. When the root developed and the apical foramen was closed, the crown would align with the contralateral tooth. This treatment, referred to as root-oriented orthodontic traction treatment, could minimize premature closure of the apical foramen due to orthodontic traction.

Our application of an adjustable active traction device, anchored on the upper first molar, ensures upward force application, counteracting displacement tendencies. This design, necessitating minimal fixation and allowing removal during meals, provides a distinct advantage over static devices like the Hass expander by not impeding the replacement of children’s teeth. Furthermore, the device’s front incorporates a gap expansion spring, facilitating concurrent traction and gap creation. This design feature effectively reduces resistance to both eruption and root development, thereby shortening treatment duration, maintaining gentle continuous force, and significantly lowering the risk of root resorption for both the impacted tooth and adjacent structures.

The academic debate persists regarding the timing of traction in relation to root development, with some suggesting it promotes periodontal tissue and alveolar bone remodeling,^[10]^ while others caution against potential root resorption risks.^[11]^ Notably, our cases demonstrate significant root development post-traction, albeit with discernible variances. Han Hu posits that inverted impacted teeth subjected to orthodontic traction exhibit a growth potential akin to that of healthy teeth.^[12]^ In an illustrative follow-up at 11 months, with the active traction device inadvertently dislodged and considering the root’s distance from the palatal cortical bone coupled with corrected eruption path and ongoing developmental potential, the decision to remove the orthodontic device was made to allow natural eruption. Regular post-operation follow-ups are imperative to monitor long-term outcomes, including periodontal health, especially given the theoretical risk of root absorption and gingival recession over time.^[13]^ Sequential CBCT images revealed continuous root development and secondary curvature, suggesting a favorable deviation from the labial cortical bone. Moreover, post-operation analysis revealed improved keratinized gingiva quality in Case 2, underscoring the importance of a tailored surgical approach.

## 4. Conclusions

This case report advocates for root-oriented orthodontic traction treatment in the management of impacted maxillary central incisors. Reducing root developmental resistance and ensuring substantial root development could leverage the inherent growth potential for optimal development and alignment, obtaining superior outcomes compared to conventional orthodontic traction.

## Acknowledgments

Thanks to the patients who presented in this case report.

## Author contributions

**Data curation:** Wei Chen.

**Funding acquisition:** Wei Zhang.

**Investigation:** Wei Chen, Jingyao Bai.

**Methodology:** Wei Chen, Wei Zhang.

**Project administration:** Wei Zhang.

**Software:** Wei Chen.

**Supervision:** Jiqin Zhang.

**Writing – original draft:** Jingyao Bai.

**Writing – review & editing:** Jiqin Zhang.
